# Interactions in the microbiome: communities of organisms and communities of genes

**DOI:** 10.1111/1574-6976.12035

**Published:** 2013-08-28

**Authors:** Eva Boon, Conor J Meehan, Chris Whidden, Dennis H-J Wong, Morgan GI Langille, Robert G Beiko

**Affiliations:** 1 Department of Biology, Dalhousie UniversityHalifax, NS, Canada; 2 Faculty of Computer Science, Dalhousie UniversityHalifax, NS, Canada; 3 Department of Biochemistry and Molecular Biology, Dalhousie UniversityHalifax, NS, Canada; 4 Faculty of Graduate Studies, Dalhousie UniversityHalifax, NS, Canada

**Keywords:** microbial communities, metagenomics, genome evolution, trait-based ecology, Black Queen Hypothesis, Public Goods Hypothesis

## Abstract

A central challenge in microbial community ecology is the delineation of appropriate units of biodiversity, which can be taxonomic, phylogenetic, or functional in nature. The term ‘community’ is applied ambiguously; in some cases, the term refers simply to a set of observed entities, while in other cases, it requires that these entities interact with one another. Microorganisms can rapidly gain and lose genes, potentially decoupling community roles from taxonomic and phylogenetic groupings. Trait-based approaches offer a useful alternative, but many traits can be defined based on gene functions, metabolic modules, and genomic properties, and the optimal set of traits to choose is often not obvious. An analysis that considers taxon assignment and traits in concert may be ideal, with the strengths of each approach offsetting the weaknesses of the other. Individual genes also merit consideration as entities in an ecological analysis, with characteristics such as diversity, turnover, and interactions modeled using genes rather than organisms as entities. We identify some promising avenues of research that are likely to yield a deeper understanding of microbial communities that shift from observation-based questions of ‘Who is there?’ and ‘What are they doing?’ to the mechanistically driven question of ‘How will they respond?’

## Introduction

Microorganisms are everywhere, but they rarely act alone. The best illustration of this fact is the ‘Great Plate Count Anomaly’ (Staley & Konopka, 1985), which claims that < 1% of all known microorganisms can be successfully cultured on their own. It is now clear that many microorganisms depend on the activity of other microorganisms to successfully grow and reproduce (Schink, 2002; Stolyar *et al*., 2007; McCutcheon & von Dohlen, 2011; Hug *et al*., 2012) via mechanisms including acquisition and exchange of metabolites (Stams, 1994; Falony *et al*., 2006; Carini *et al*., 2012). The diversity of microbiomes is being explored using surveys that draw on hundreds or thousands of samples (Caporaso *et al*., 2011; Human Microbiome Project Consortium, 2012; Larsen *et al*., 2012) and controlled experiments (McNulty *et al*., 2011; Lawley *et al*., 2012; Xie *et al*., 2012; Badri *et al*., 2013; Smith *et al*., 2013), with rapid genetic assessment techniques providing much of the evidence for taxonomic and functional diversity. Long-standing questions in microbial ecology such as whether ‘everything is everywhere, but the environment selects’ (Baas Becking, 1934; de Wit & Bouvier, 2006) can now be tested in fine detail by examining the geographic and habitat distributions of microorganisms (Martiny *et al*., 2006). The web of microbial interactions spans all taxonomic ranks, from strain to superkingdom, and underscores the need for community-centric approaches to understanding microbial diversity (Zarraonaindia *et al*., 2013).

Microbial ecology has benefited greatly from the adaptation of theories and methods developed initially for multicellular organisms (Prosser *et al*., 2007). Hypotheses about the distribution of microorganisms can be tested biogeographically by contrasting biotic similarity with habitat distances and geographic distance (Martiny *et al*., 2006), using approaches originally developed with macroorganisms in mind. Microbial community ecology has drawn heavily upon classical approaches, in particular the representation of biodiversity in terms of the entities (e.g. species) that are present, often with additional information about the relative abundance of different entities. Although species are commonly thought of as ecological units and thus the most natural entities to count and search for interactions, several reasons motivate the use of other units to quantify biodiversity. Larger taxonomic groups may be of interest because they share one or more important attributes: Class *Amphibia* is seen as a leading indicator of a general decline in biodiversity, in part due to their sensitivity to disturbances in both terrestrial and aquatic habitats (Collins & Crump, 2009), while the balance between *Bacteroidetes* and *Firmicutes* is sometimes treated as a defining feature of the human gut microbiota (Ley *et al*., 2006; Mariat *et al*., 2009), even though both bacterial phyla contain a wide range of organisms with distinct ecological roles (Qin *et al*., 2012). While community ecology considers interactions among entities, the inference of interactions can depend critically on the level at which entities are defined. Models can be used to predict the impact of interactions on expected abundances over time, and web and network structures can capture a complete range of possible pairwise interactions between community members (May, 1973; Menge, 1995; Faust *et al*., 2012; Larsen *et al*., 2012). Microbial community ecology has the potential to identify key interactions between microorganisms, with a wide range of important applications in health and the environment (Preidis & Versalovic, 2009; Bakker *et al*., 2012; Costello *et al*., 2012).

Success in applying well-developed ecological theories to microorganisms has been achieved in spite of the obvious differences between microorganisms and multicellular organisms. Differences in size, dispersal, dormancy regimes, and growth and reproduction may not prohibit application of the same quantitative techniques to both single-celled and multicellular organisms. However, genome-scale data have given an evolutionary context to the phylogeny function disconnect in microorganisms, particularly bacteria, which has been known for decades (Cowan, 1955; Floodgate, 1962), in the process spawning or reigniting debates about microbial evolution, taxonomy, and the microbial species (Gevers *et al*., 2005; Konstantinidis & Tiedje, 2005; Bapteste & Boucher, 2008; Doolittle & Zhaxybayeva, 2009). Although a unifying species concept is not needed for ecological analysis, a sound rationale and clear approach (or set of approaches) to define ‘units’ is. The use of uniform taxonomic or phylogenetic thresholds may fail to adequately delineate ecologically cohesive units, especially in microorganisms whose genomes can change rapidly through gene loss, gene duplication, and the acquisition of genes from distant lineages via lateral gene transfer (LGT).

When considering the nature of microbial communities, especially in the inference of interactions that determine community structure, we must assess the potential impact of microbial evolutionary processes on the entities that constitute these communities. In this article, we review several aspects of communities and community interactions, starting with the definition of ‘community’ itself. We then consider different approaches used to define the entities in a potential community, in particular the broad range of trait-based approaches that have recently been developed and applied in different settings. Because traits are ultimately conferred by an organism’s genes, we then examine the evolutionary dynamics of these genes, culminating in two recent hypotheses (McInerney *et al*., 2011; Morris *et al*., 2012) that address potential impacts of gene gain and loss on microbial interactions. The dynamic movement of genes through microbial lineages and communities suggests that genes themselves may be treated as valid ecological entities, and we propose a metacommunity framework for the analysis of gene distributions. Finally, we consider the ecological unit definitions that are currently in use, and we highlight how these definitions might be augmented by explicit consideration of interactions and evolutionary models in experimental and analytical techniques.

## Defining and measuring communities and microbiomes

The term ‘community’ and the related term ‘assemblage’ have long been used in ecology, but their definitions are both fluid and controversial (e.g. Ricklefs, 2008). For example, Fauth *et al*. (1996) uses assemblage to define ‘phylogenetically related groups within a community’ with a community described as a ‘collection of species occurring in the same place at the same time’. Cornell & Lawton (1992) distinguish ‘interactive’ from ‘noninteractive’ communities based on the presence or absence of biotic interactions. In a similar fashion, Konopka (2009) defines communities as ‘multispecies assemblages, in which organisms live together in a contiguous environment and interact with each other’. We adopt this latter definition of ‘community’ while recognizing that it is neither comprehensive nor universally accepted (e.g. Zarraonaindia *et al*., 2013). We consider an assemblage to be the set of species (or, more generally, taxa) that are inferred to be in a given place at a given time, based on evidence from morphology or sequence data. Thus, we treat ‘community’ as a refinement of ‘assemblage’, with the additional proviso that taxa interact with one another. These definitions usefully distinguish observations (assemblages) from testable hypotheses (communities).

The definition of ‘microbiome’ has a shorter, but equally tortuous history. Although there is consensus that the term was first coined by Joshua Lederberg in 2000 or 2001, confusion arises because the term can be read as ‘micro-biome’ (the set of resident microorganisms and associated abiotic factors) or ‘microbi-ome’ (the complete set of genetic information associated with a set of microorganisms). The definition was given by Lederberg & McCray (2001) as ‘…the ecological community of commensal, symbiotic, and pathogenic microorganisms that literally share our body space’, and has expanded from its initial application to human-associated microorganisms (Relman, 2002; Turnbaugh *et al*., 2007) to encompass microorganisms in any setting (Gilbert *et al*., 2010b). Host-associated microbiota or microbial communities are frequently described as symbionts (Mandel, 2010; Ballal *et al*., 2011), but this is almost certainly not true for all microorganisms observed in a healthy human. As described above, the existence and nature of interactions among microorganisms and their host represent a hypothesis to be tested. We therefore favor an observation-based definition of the microbiome as the set of microorganisms and their genomes in a particular environment, without any requirement for ecological interactions. Whether one or more communities exist within a given microbiome is a matter for further investigation.

### Community interactions

Interactions have been treated as a central feature of communities since the early 1900s, but how these interactions are interpreted has changed many times. Clements (1916) described succession in plant communities, or ‘seres’ as he called them, as a series of associations from pioneer to climax communities. The development of a sere was likened to that of an organism. (Gleason 1926) articulated what might be viewed as a first null model of community interactions: ‘Are we not justified in coming to the general conclusion, far removed from the prevailing opinion, that an association is not an organism, scarcely even a vegetational unit, but merely a coincidence?’ Elton (1927), like Clements, also drew the analogy of community and organism when he wrote: ‘animal associations, or better, animal communities, … are not mere assemblages of species living together, but form closely knit communities or societies comparable to our own’. Many authors viewed interacting organisms in a community as constituting a ‘complex organism’ with emergent properties, as summarized by Phillips (1935). It was only later in the 1950s and the 1960s that the idea of communities as organisms lost its popularity, and so-called ‘species–individualistic’ models gained more popularity (Whittaker, 1967; Ricklefs, 2008). The precise definition of community in any given study is explicitly or implicitly determined by the investigator’s choice of experimental techniques and analytical tools: As Konopka (2009) states, ‘The practical delineation of “community” may then reflect the interests of the ecologist rather than any inherent characteristics’.

**Figure 1:**
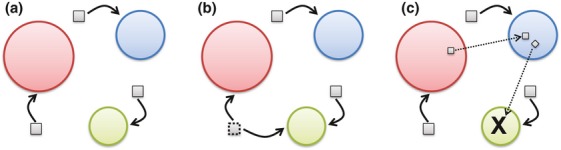
Conceptual representation of communities: (a) no interactions (i.e. a neutral community model), (b) indirect interactions (competition for a resource), (c) direct interactions (cross-feeding and targeted killing). Circles represent individuals, squares indicate a resource, and diamonds indicate a toxic substance.

A spectrum of degrees of interaction is conceivable (Fig. 1). At one end of this spectrum lies a null interaction model similar to that articulated by Gleason, with distinct organisms found in a particular setting being mutually oblivious or interacting only in trivial ways. In this scenario, the presence of one organism has no effect on the viability of another, which corresponds to the ‘assemblages’ defined previously. At the other end of the spectrum would be coevolved obligate interspecies interactions that are mutually beneficial and highly specific and that bind species so tightly that independent existence, or association with alternative species, is no longer possible. Between these two extremes lie a range of interaction types, from protagonistic (mutualism) to benign (commensalism) to antagonistic (e.g. predation or parasitism), with each interaction type varying from obligate to facultative (Little *et al*., 2008). Dependencies can be based on metabolic interactions, as in cross-feeding or pathway completion where microorganisms engage in reciprocal or nonreciprocal exchange of metabolites (Helling *et al*., 1987; Wintermute & Silver, 2010).

**Figure 2:**
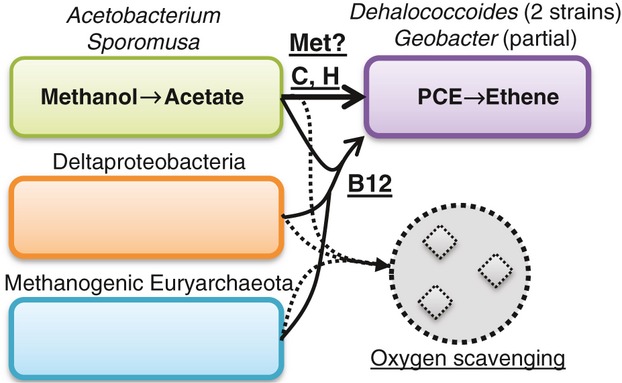
Interactions supporting the growth and metabolism of the key dehalogenating organisms *Dehalococcoides* and *Geobacter* via metabolite provision (solid arrows) and detoxification via oxygen scavenging (dashed arrows) in the KB-1 mixed culture. Key metabolites and functions provided by other members of the community are underlined. Met, methionine, PCE, perchlorinated ethene.

Extreme examples of tight interactions include the association between the endosymbiotic bacteria *Candidatus* ‘Moranella endobia’ and *Candidatus* ‘Tremblaya princeps’, which live inside the cells of the mealybug *Planococcus citri* (McCutcheon & von Dohlen, 2011). In this system, synthesis of several amino acids including phenylalanine, arginine, and isoleucine appears to depend on exchange of pathway intermediates and successive reactions that are carried out by different community members. Less dramatic are systems in which microorganisms depend on pathway end products that must be synthesized by others. Many organisms within the *Dehalococcoides* genus perform reductive dehalogenation, a process of great importance in bioremediation, for example in the commercially successful KB-1 mixed culture (Duhamel *et al*., 2002; Smidt & de Vos, 2004). However, to be cultured axenically, *Dehalococcoides* requires a specialized reduced medium containing vitamin B12 (Löffler *et al*., 2012); despite significant efforts, *Dehalococcoides* grows much more slowly and to lower cell density in axenic culture compared with mixed cultures. Metagenomic analysis suggests dependencies on other community members for cofactor precursors and possibly methionine (Hug *et al*., 2012; see Fig. 2). Dependencies can also be indirect through modification of the surrounding medium, such as the reliance on other organisms to detoxify or sequester harmful compounds in the environment (Morris *et al*., 2012). In addition to the specific dependencies mentioned above, *Dehalococcoides* strains found in many mixed cultures also depend on other community members for oxygen scavenging (Hug *et al*., 2012). Negative interactions have been observed between microorganisms at every degree of taxonomic divergence. These can be indirect, based on competition for a particular resource or secretion of a broadly toxic compound. Direct negative interactions involve the targeting of a potential competitor using inhibitory compounds such as antibiotics or bacteriocins, parasitism, or predation (Hibbing *et al*., 2010).

### Describing the structure of assemblages and communities

Characterizing the distribution (presence and relative abundance) of an assemblage of microorganisms is a precondition for testing community structure. The assessment of microbial diversity has shifted from primarily culture-based methods to approaches that make use of rapidly improving DNA sequencing technology. Often, a marker gene such as the 16S ribosomal RNA gene (referred to as 16S henceforth) is targeted and sequenced to give an indication of the taxonomic diversity within a given sample (Ward *et al*., 1990; Amann *et al*., 1995). There are several drawbacks to such single-gene studies. First, the plasticity of prokaryotic genomes means that the use of 16S as an indicator of diversity often masks many of the differentiating traits between closely related organisms (Dobrindt & Hacker, 2001; Medini *et al*., 2005). Therefore, the interactions, and the set of distinct entities in a sample, can be difficult to interpret from 16S studies alone. Second, a community is often defined by a stable species composition, but such stability is not always found in microbial settings (Turnbaugh *et al*., 2009; Booijink *et al*., 2010; Caporaso *et al*., 2011; Human Microbiome Project Consortium, 2012). As such, taxonomy-centric definitions may not be sufficient to yield an adequate understanding of microbial ecology (Shade & Handelsman, 2012). An alternative to marker gene studies is environmental whole-genome shotgun (WGS) sequencing as pioneered by such as Venter *et al*. (2004) and Tyson *et al*. (2004)’s studies to reveal a metagenome (Handelsman *et al*., 1998). The resulting set of DNA sequence reads can potentially cover the entire genomes of the sampled microorganisms (given sufficient sequencing effort), not just a given marker gene. Such an approach can reveal the functional complement of a given sample and suggest interactions between members based on such functions. However, assembly and assignment of function and taxonomy to metagenomic sequences is a complicated task that often generates a multitude of low-confidence predictions (Prakash & Taylor, 2012) and ambiguities about which sequences may have originated in the same organism. This leads to difficulty in creating a complete consensus of community function and diversity and linking these two aspects to each other.

**Figure 3:**
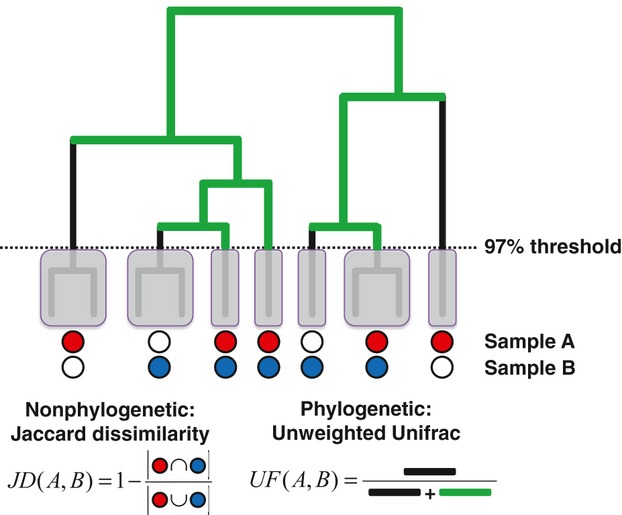
The application of nonphylogenetic and phylogenetic diversity measures to two samples of microorganisms. OTUs at 97% present in sample A and sample B are shown with red and blue circles, respectively. OTUs absent from samples are shown with white circles. Black edges in the tree have leaves from only one of the two samples as descendants, while green edges cover both samples. The calculation of two unweighted (qualitative) measures of community dissimilarity is indicated at the bottom.

Taxonomic diversity has historically been expressed in many ways. Assemblages can be considered in terms of the presence or relative abundance of different discretely defined groups, which are circumscribed using either a taxonomic ranking and naming scheme or an assessed degree of genetic relatedness. Measures such as species richness, Shannon diversity, Jaccard dissimilarity, and Bray–Curtis dissimilarity have been applied to microbial communities to assess the impact of different habitat types on biodiversity, by using taxonomy (e.g. the *Bacteroidetes*/*Firmicutes* ratio) or by defining operational taxonomic units (OTUs: Ehrlich & Holm, 1962; Sokal & Sneath, 1963) based on the similarity of marker genes such as 16S (Schloss & Handelsman, 2005; Fig. 3A). Phylogenetic diversity considers the relatedness of different lineages, based on the underlying assumption that phylogenetic relatedness between taxa should correlate with ecological similarity (Martin, 2002). These diversity measures typically quantify the extent to which branches in a rooted phylogenetic tree are unique to one sample or the other, or common to both (Fig. 3B). Weighting by relatedness may give more biologically relevant interpretations of diversity, and phylogenetic diversity measures have gained widespread use in microbial community analysis (Kuczynski *et al*., 2010; Parks & Beiko, 2013).

Much care is warranted in the choice of the relevant level(s) of diversity. If our focus is the community, it might be reasonable to choose the species boundary as the main delimiter of diversity, because this boundary typically distinguishes the study of populations from that of communities (Prosser *et al*., 2007). The most widely cited definition of microbial species is that of Dykhuizen & Green (1991), which defines recombination as the key driver of species cohesion and in fact requires the consideration and comparison of multiple gene trees. However, there is still no definition that refutes Cohan & Perry (2007): ‘However, no sequence-based OTU proposed either by systematists or by ecologists appears to correspond to the fundamental units of bacterial ecology’.

## Taxonomy or traits as the basis for communities?

The focus of microbial ecology on taxonomically and phylogenetically cohesive groups is shared with macroorganism-based ecology and facilitated by the tractability of taxonomic marker genes to sampling and analysis, as well as the availability of large phylogenetic databases such as the Ribosomal Database Project (Maidak *et al*., 2001) and GreenGenes (DeSantis *et al*., 2006) for mapping purposes. By contrast, trait-based ecology (Hutchinson, 1957; Green *et al*., 2008) represents organisms in terms of functional properties that may impact their fitness in a given habitat (i.e. *functional traits*) and their responses to disturbance. The key to trait-based ecology is the mapping of species information into a *functional space* that expresses the similarity of morphological, behavioral, or biochemical traits that can influence the ability to occupy particular niches (Hutchinson, 1957; Mouillot *et al*., 2013); this type of approach recalls microbial classification schemes developed before the advent of DNA sequencing (Sapp, 2005). Because traits mediate the interactions among organisms and between organisms and the environment, many have argued that trait-based approaches are more relevant to community analysis than taxonomic or phylogenetic attributes (McGill *et al*., 2006; Violle *et al*., 2007). Although environmental properties will limit the types of organisms that can occupy a particular habitat, the taxa that can potentially occupy that habitat need not be closely related to one another. In some cases, occupancy may be driven by a stochastic ‘lottery’ process (Sale, 1978; Burke *et al*., 2011a) that need not respect species boundaries or even be constrained to a given clade in a tree.

Taxonomic and phylogenetic approaches to community analysis impose either a discrete or a hierarchical classification of entities (Mishler & Donoghue, 1982; Cohan & Perry, 2007). Phylogenetic approaches require units to be monophyletic, whereas named species would typically be monophyletic or paraphyletic in practice if not by definition. Trait distributions set aside the expectation of monophyly or paraphyly of units and need not respect clusters or lineages. The majority of an organism’s traits will not respect a species boundary, however defined, and will either be shared with others outside of its species group, vary within its species group, or both. These types of variations motivated Van Valen (1976) to propose the ecological species concept as opposed to the biological (or ‘reproductive’) species concept of Mayr (1942). Convergent evolution can lead distantly related organisms into the same region of ‘trait space’, either via convergent morphological evolution (Kocher *et al*., 1993; McNab, 2009) or via independent invention of similar systems such as C4 photosynthesis (Sage *et al*., 2011). A striking example of trait convergence was reported by Fan *et al*. (2012), who examined the phylogenetic and functional diversity of microorganisms associated with six different types of sponge. The phylogenetic structure was consistent in replicated samples from each sponge and differed markedly between sponge species. However, a range of metabolic and cellular traits including denitrification and cofactor synthesis were consistently enriched relative to seawater samples.

Complementing convergence is the possibility of rapid genotypic and phenotypic divergence, even among organisms that satisfy criteria for membership in the same species. The impact of this divergence has been well documented in many plant species. For instance, genetic variation within *Populus angustifolia* affects resistance to aphids and influences a wider community of associated macro- and microorganisms (Bailey *et al*., 2005, 2006; Whitham *et al*., 2008). While a species-based analysis might capture some aspects of the community in such a case, the key genetic distinctions within *P. angustifolia* would be completely lost, impeding an understanding of community function.

### Traits in microbial ecology

Given the extensively documented genomic and ecological variation in microorganisms, the limitations of taxonomic and phylogenetic approaches will be more acute in these organisms. This motivates the application of trait-based approaches as an alternative (Green *et al*., 2008). Not surprisingly, the list of traits considered is dominated by those that can be assayed using genomic and related approaches, including sequence dissimilarity, ribosomal copy number, and genome size. Given a metagenome sample that has been functionally annotated using a reference database, it is possible to examine the profile of many or all functional categories of proteins, as has been performed by DeLong *et al*. (2006) in a depth transect of the ocean, Raes *et al*. (2011) for a series of sites from the Global Ocean Sampling expedition (Rusch *et al*., 2007), and others. Because broad functional summaries may miss important differences within groups that drive ecological differences, approaches that target a subset of functions such as nitrogen cycling in soil (Bru *et al*., 2010), butyrate production in the human gut (Van den Abbeele *et al*., 2013), or membrane proteins in different ocean habitats (Patel *et al*., 2010) can be more informative about the relationship between traits and habitat type. Although genomes and metagenomes give a detailed cross section of the functional potential of an organism or a community, it is the phenotypic traits that interact directly with the environment, and these may provide more relevant information in a community analysis (Kim *et al*., 2009; Gudelj *et al*., 2010). Phenotypic traits determined by one or a few genes, such as toxin resistance or degradation of a relatively simple carbohydrate, may often be predictable from genotype. However, complex phenotypes such as cell shape, and traits where subtle sequence differences can lead to drastic ecological consequences (such as peptide receptors: Geisinger *et al*., 2008), will require either more sophisticated modeling or direct experimental characterization of the phenotype of interest (Whitworth, 2008).

The extensive phenotypic diversity within many named species of microorganisms that satisfy the typical criteria for species membership (i.e. 70% DNA–DNA hybridization or 97% identity of the small-subunit ribosomal RNA gene) has been well documented: As Cohan & Perry (2007) state, ‘…the recognized “species” of bacterial systematics frequently contain a diversity of populations that are distinct in their biochemistry, physiology, genome content and ecology; classifying an unknown organism to its species thus tells us only vaguely about the organism’s way of life’. This assertion has been shown to be true for oceanic microorganisms such as the remarkably diverse *Prochlorococcus marinus* (Martiny *et al*., 2009) and SAR11 (Morris *et al*., 2002) and for host-associated organisms such as *Lactobacillus plantarum* (Siezen *et al*., 2010) and *Escherichia coli* (Souza *et al*., 1999; Welch *et al*., 2002). This diversity highlights the promise of trait-based analysis, but the application of traits in microbial ecology requires a thorough understanding of their genetic underpinnings and the evolutionary processes that generate and sustain them.

## Genome evolution and microbial interactions

Processes at the genome level influence the evolution of microbial traits and the emergence of microbial community structure. If we are to consider trait-based approaches to microbial ecology, then it is essential to understand the evolutionary dynamics of these traits. The following insights gained by comparative analysis of sequenced genomes offer a useful framework to understand why trait-based approaches may be complementary to those based on phylogenetic markers.

### Gene loss

**Figure 4:**
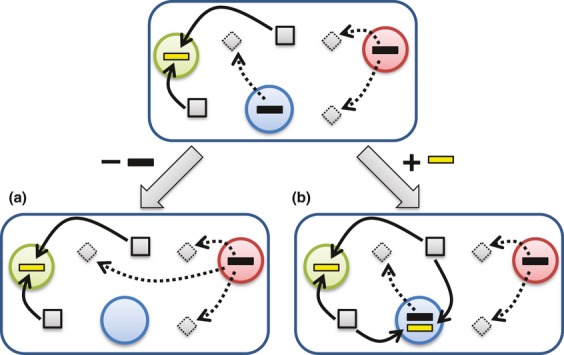
Contrasting two modes of bacterial evolution that modify the genotype and ecological role of a microorganism. The top shows an assemblage of three organisms colored green, blue, and red. Squares indicate a resource that is taken up and metabolized by the cell (yellow bars), and diamonds indicate a toxic substance that is metabolized by the secretion of enzymes from producing cells (black bars). (a) Gene loss via the BQH: Because the red organism can metabolize the toxic substance, the blue organism gains an energetic advantage, by not expressing (and eventually, no longer encoding, due to gene loss) the detoxification pathway. The blue organism then becomes dependent on other members of the community to carry out this process. (b) Gain of function according to the Public Goods Hypothesis: The blue organism acquires a gene or pathway from the green organism via LGT and emerges as a competitor for the resource.

At the heart of microbial evolution is a process of genome streamlining that rapidly discards genetic material that is not under selection, a process that appears to carry an advantage to the organism (Lynch, 2006; Koskiniemi *et al*., 2012). The most striking examples of this process are seen in genomes that are currently in niche transition or have recently undergone such transitions. *Mycobacterium leprae* exemplifies this process: The organism resides in macrophages, but bears residual evidence of a less constrained lifestyle, with more than 1000 pseudogenes providing clear evidence of recent losses of respiration, catabolic, and other pathways (Cole *et al*., 2001). In such cases, many genes are lost because functions such as host defense or carbohydrate metabolism are no longer needed. Some amount of gene loss can be offset by increasing the density of the interaction network among the proteins that remain (Kelkar & Ochman, 2013), but gene loss may also arise when a resource-intensive function can be performed by one or more other members of the community. The Black Queen Hypothesis (BQH) of Morris *et al*. (2012) considers the trade-off between the potential cost of losing one or more genes encoding a particular function and the benefit of offloading the resource burden associated with this function onto another member of the community. However, specialization due to loss of function (Fig. 4a) creates a dependency on other community members for that function and therefore requires a certain degree of community stability, potentially in combination with dormancy when key conditions for growth and reproduction – including the presence of essential ecological partners – are not met (Lennon & Jones, 2011).

### Lateral gene transfer

Complementing gene loss in microorganisms is LGT, a process of gene gain that can rapidly reshape the ecological capacity of a lineage (Fig. 4b). LGT offers a path by which organisms can recover from the specialization and streamlining that accompany gene loss. While estimates of the extent of LGT vary depending on the microorganisms studied, the analytical methods used, and the way in which transfer events are counted (Beiko *et al*., 2005; Ge *et al*., 2005; Kunin *et al*., 2005; Ragan *et al*., 2006; Dagan *et al*., 2008), there is no question that LGT is widespread in microorganisms and appears to play a major role in the generation of functional novelty, at least in the short term, than gene duplication (Treangen & Rocha, 2011). LGT clearly violates assumptions of treelike descent and speciation, introducing connections between distant microbial phyla and even between domains (Puigbò *et al*., 2009; Dodsworth *et al*., 2010; Beiko, 2011; Clarke *et al*., 2013). The mechanisms that enable LGT may not have gene transfer as their primary purpose in microbial cells and may instead serve primarily as agents of resource acquisition and DNA repair (Redfield, 2001) and as mechanisms by which selfish elements propagate themselves. A considerable amount of LGT may conform to the neutral theory of evolution in that many gene acquisitions are deleterious (Skippington & Ragan, 2011) or selectively neutral. Although most genome-scale analyses tend to focus on annotated protein-coding genes, more thorough analysis has shown that many genomes contain the remnants of acquired genes that were presumably neutral and are currently undergoing decay and loss (Hao & Golding, 2006). While LGT events often confer no benefit to the recipient organism, LGT-enabling mechanisms are clearly exploited by organisms in some settings where LGT may be beneficial: For example, loss of DNA repair systems can lead to a ‘LGT mutator’ phenotype (Denamur *et al*., 2000). Recently, Johnston *et al*. (2013) demonstrated that some strains of *Streptococcus pneumoniae* possess methylases that can protect internalized DNA from cleavage by restriction enzymes; the authors argue that this system facilitates the exchange of pathogenicity islands and other material among closely related strains. Further supporting the potentially beneficial role of LGT is the demonstration that acquired genes have been successfully integrated into the host’s regulatory and metabolic networks (Pál *et al*., 2005; Lercher & Pál, 2008).

What role does LGT have in establishing and maintaining communities? Biofilms are a primary example of a microbial community driven by LGT. Biofilms usually comprise more than one named species (Wolcott *et al*., 2013) and have been extensively studied in many settings. Recently, the study of medically important biofilms has generated new hypotheses about the role of LGT in communities. Biofilms in chronic infections can persist by subverting host cellular pathways (Kim *et al*., 2010) to, for example, prevent apoptosis, rather than expressing planktonic virulence factors such as toxins. Microorganisms in biofilms undergo rapid LGT and are often genetically distinct, as exemplified by the fact that 10% of the genes in clinical isolates of *Haemophilus influenzae* are unique as compared to sequenced laboratory strains (Shen *et al*., 2006) and the demonstration of *in vivo* LGT over time within multiple strains of *S. pneumoniae* infecting a single pediatric patient (Hiller *et al*., 2010). The distributed genome hypothesis (Ehrlich, 2001; Ehrlich *et al*., 2010) argues that constituents of some bacterial biofilms collectively possess a community genome that evolves through rapid and focused transfer. This hypothesis suggests that biofilm communities can outcompete host defenses by continuously generating a cloud of novel strains and gene combinations through LGT. Further, this gene acquisition can be regulated through quorum sensing, possibly even between different species (Antonova & Hammer, 2011; Zhu & Li, 2012). A biofilm is a mature example of a community where limited barriers to LGT, distribution of tasks, and close proximity provide incentives to cooperate and maintain the biofilm. The far-reaching level of functional integration has even led some authors to propose that the biofilm itself is the biological individual (Ereshefsky & Pedroso, 2013) based on both the degree of integration and the similar way in which community members respond to the environment (i.e. a ‘unitary response’ *sensu* Hull, 1980). Under other definitions of the individual that consider independence of replication, a biofilm is a microbial community with obligate and specific interactions that include even the timed exchange of genetic material.

Outside of biofilms, researchers have tried to understand why obligate associations such as cross-feeding emerge: Pfeiffer & Bonhoeffer (2004) used chemostat simulations to highlight the potential benefits of cross-feeding in ATP production and maintaining low concentrations of enzymes and intermediates, while Zhuang *et al*. (2011) pointed to membrane space as a potential limiting factor in respiration. The emergence of associations is likely mediated by a number of forces including habitat stability, physiological constraints, and the costs of carrying out reactions. Fan *et al*. (2012) identified a wide range of different mobile genetic elements (MGEs) in the sponge-associated microorganisms they studied, and suggested that these elements (particularly transposases) might play a role in adaptation of community members to a common host and in disruption of genes that are no longer needed due to the formation of stable associations.

‘Cheating’ microbial strains – microbial strains that have lost important community functions such as quorum sensing, but still acquire resources from other community members (Diggle *et al*., 2007) – also illuminate the role of LGT in community development. Maintaining functions important to the community on MGEs (e.g. plasmids) or using mechanisms such as quorum sensing to restrict LGT may penalize cheating strains by forcing them to reacquire the lost function (Smith, 2001) or to avoid LGT and be outcompeted (McGinty *et al*., 2011). Cooperative genes and genes that confer virulence are overrepresented in MGEs (Nogueira *et al*., 2009), among other traits (Rankin *et al*., 2011), lending support to this theory. In a metagenomic study of a contaminated groundwater community, Hemme *et al*. (2010) found evidence for transfer of genes conferring resistance to many contaminants including mercury and acetone. Harrison & Brockhurst (2012) alternatively suggest that LGT mediated by plasmids is a process of coevolution between chromosomal and plasmid genomes that prevents beneficial genes from simply being absorbed into the chromosomal genome, which would lead to plasmid loss via purifying selection.

LGT and other processes call into the question the utility of phylogenetic cohesion as an exclusive criterion for defining ecological units. If genes can be readily acquired via LGT, then they might be considered a common resource accessible to microorganisms. The Public Goods Hypothesis (PGH) of McInerney *et al*. (2011) claims that genes are public goods if they satisfy the *nonrival* and *nonexcludable* criteria: ‘A good is nonrival if the consumption of the good by one individual does not reduce the availability of that good for another individual. A good is nonexcludable if it is impossible or at least very difficult to exclude the good from being available to everybody’. In treating protein-coding genes, which satisfy these criteria, as the resource, the PGH inverts the BQH to focus on gene acquisition rather than on gene loss as an evolutionary opportunity. Both models offer competitive advantages to organisms that focus their resources on tasks that are not effectively provided by other community members, but the two models are driven by different evolutionary processes (Fig. 4). The interplay of gene acquisition and loss – coupled with other methods of generating novelty such as point mutations and gene duplications – creates an evolutionary and ecological dynamic that may invalidate traditional community models.

### The relevance of genome streamlining and expansion

The fundamental processes of gene loss and gene gain via LGT impact the evolution of microbial lineages and communities, with global consequences. In the ocean, the ubiquitously distributed and heterotrophic SAR11 group includes an enormous diversity of phylotypes and strains, with wide variation in latitudinal patterns of occurrence and possibly the largest effective population size of any bacterial group (Morris *et al*., 2002). Genomes of this group are highly streamlined, with extremely short intergenic spacers and a genome size of *c*. 1.3 Mb (Giovannoni *et al*., 2005). Large amounts of genome-level variation are present in some regions and in association with particular functions, especially membrane proteins (Wilhelm *et al*., 2007; Brown *et al*., 2012). This variation is supported by the observation of very high levels of homologous recombination that disrupt clonal relationships within the group (Vergin *et al*., 2007), to the point where even ribosomal operons of SAR11 show evidence for homologous recombination (Suzuki *et al*., 2001). The exchange of genetic material is not limited only to members of the group, as some genes appear to have been acquired from groups such as cyanobacteria (Gilbert *et al*., 2008; Viklund *et al*., 2012). A crucial property of this group is the absence of many DNA repair genes such as *mut*LS that are found in the alphaproteobacterial relatives of SAR11. Because the *mut*LS complex ordinarily prevents homologous recombination of divergent sequences, its absence from the SAR11 group appears to be responsible for the observed elevated rate of mutation and gene gain and loss (Viklund *et al*., 2012). Although SAR11 is an especially dramatic example of the opposing forces of genome reduction and gene gain via LGT, it is by no means unique: A primary case study underpinning the BQH is the dependence of the abundant marine photoautotroph *P*. *marinus* on other members of its community for peroxide decontamination with many catalase-positive organisms from a wide range of taxonomic groups able to provide this function (Morris *et al*., 2011). This dependency of *P. marinus* recalls that of *Dehalococcoides* described above and in Fig. 2. As microbial communities continue to be explored using laboratory experiments and genetic profiling, many more examples will be discovered.

The ‘use it or lose it’ theme of microbial evolution does not preclude the emergence of relatively large genomes and generalist microorganisms. In contrast with the genome streamlining and ecological partitioning seen in SAR11 and elsewhere, many microorganisms have genomes > 6 megabases in size. These genomes tend to be enriched in genes for regulation, secondary metabolism, and signal transduction (Konstantinidis & Tiedje, 2004; Koonin & Wolf, 2008), with many of these genes acquired via LGT (Cordero & Hogeweg, 2009). The largest prokaryotic genome sequenced to date is that of *Sorangium cellulosum* strain So ce56 (Schneiker *et al*., 2007), a standout in the already large myxobacterial group with a genome in excess of 13 megabases of DNA. Its ecology and complement of functional genes are far from being completely elucidated: More than 4400 genes had no assignable function from homology, and 3248 were proper ‘orphans’ with no detectable homologs in any other genome. Among genes with inferred functions, many are associated with secondary metabolite production, cell movement, sophisticated social behaviors including quorum sensing and fruiting body formation, and complex carbohydrate degradation. Evolutionary theory suggests that effective population sizes must be small, and the role of drift must be substantial, to allow genomes to grow large (Lynch, 2006). Gained genes must be advantageous in the organism’s niche or niches, with only ‘fastidiously growing prokaryotes that inhabit complex, variable environments’ (Koonin & Wolf, 2008) likely to acquire and retain large numbers of new genes. Certainly many (but not all) of the largest microbial genomes are from soil-associated organisms such as the myxobacteria. The genus *Pseudomonas* contains more than 200 named species of environmental organisms and pathogens, with genomes typically in the range 5–7 MB, many of which can occupy multiple habitats thanks to gene duplication and LGT (Shen *et al*., 2006; Holloway & Beiko, 2010; Loper *et al*., 2012). The increased production of secondary metabolites may point to interactions with other microorganisms in a habitat, including negative interactions where the metabolites produced are intended to keep competitors at bay (Borriello, 1990; Cotter *et al*., 2013).

## Mapping genes and molecular systems into a community framework

Having outlined ideas about ecological communities and the evolutionary processes in microorganisms that complicate the relationship between organismal phylogeny and function, we now consider current taxonomic and functional knowledge about microbial communities. These insights will allow us to develop ideas that fuse these aspects of microbial evolution and ecology into potentially new modes of analysis.

### The search for a taxonomically defined ‘core’ microbiome

Ecological overlap or equivalence may be at the root of the frequently observed taxonomic differences among samples collected from the same or similar habitats. The most compelling example of this is the absence of a persistent ‘core’ microbiome in many human organs. Huse *et al*. (2012) examined the distribution of OTUs defined using a 97% identity threshold for different variable regions of the 16S. Oral and stool samples yielded a small number of OTUs that were ubiquitous or nearly so, although these were not necessarily abundant in all samples. Conversely, no OTUs were ubiquitous in many of the vaginal locations sampled, refuting the idea of a ‘core’ vaginal microbiome. Even OTUs that were ubiquitous in oral or stool samples showed differentiation among samples at higher thresholds of sequence identity, suggesting that important differences were being masked at the 97% identity level. Nemergut *et al*. (2011) examined the distribution of OTUs in different habitats such as soils, lakewater, and saline sediments and found that no OTU was ubiquitous in any habitat even when the sequence identity threshold was set as low as 89%. Deep sequencing of a marine sample (Gibbons *et al*., 2013) produced significant overlap with OTUs from a range of marine habitats, and the authors suggested that sufficiently deep sequencing at one site would reveal a ‘seed bank’ that encompasses all marine OTUs.

Several causes could contribute to the apparent lack of a ‘core’ in the many habitats examined, beyond the sampling limitations probed by Gibbons *et al*. (2013). Dispersal limitation and biogeography may play a role (Hanson *et al*., 2012), with groups such as *Pseudomonas* (Cho & Tiedje, 2000) and *Burkholderia* (Pearson *et al*., 2009), showing strong evidence of spatial structuring. Habitat definitions such as ‘soil’ and ‘gut’ are clearly too broad, as soil microbial diversity is strongly influenced by pH (Fierer & Jackson, 2006) and microhabitat (Carson *et al*., 2009; Dennis *et al*., 2009; Reim *et al*., 2012), and the composition of gut microbiota appears to strongly depend on factors such as diet (Muegge *et al*., 2011; Wu *et al*., 2011; Claesson *et al*., 2012) and the section of the gut that is sampled (Stearns *et al*., 2011). Although there is no core ‘gut’ microbiome, there may yet be a core ‘healthy transverse colon with high protein and animal fat inputs’ microbiome. Succession may also play a role, as seen for instance in the colonization of dental plaque: The same site can be occupied by ‘early’ or ‘late’ communities that emerge following a disturbance (Human Microbiome Project Consortium, 2012; Teles *et al*., 2012). Succession was also observed in the multiyear fermentation of American coolship ale, which shows a reproducible pattern in bacteria and yeast species (Bokulich *et al*., 2012). Finally, the lack of a core may reflect different outcomes of lottery processes as previously described, with observed assemblages reflecting different initial colonization events, where the first established organisms potentially structure the remainder of a community. The existence of positively correlated groups of lineages such as the ‘coabundance groups’ defined by Claesson *et al*. (2012) and groups of organisms identified in network analysis (Steele *et al*., 2011; Faust *et al*., 2012; Friedman & Alm, 2012) does not distinguish between these alternative scenarios. It does, however, suggest that the members of these groups either interact positively with one another and constitute a real community or interact in similar ways with the environment such that all are favored in the same conditions. The observed patterns also support the idea that taxonomic and phylogenetic approaches alone may be insufficient to understand the microbial ecology of a particular habitat (Shade & Handelsman, 2012).

### Functional traits in microbial assemblages and communities

If a taxonomic or phylogenetic view fails to resolve a consistent set of community properties, trait-based approaches might yield more coherent results. The ecotype model of Cohan (2002, 2006) retains the requirement that entities constitute clades, but provides a very useful working notion of a set of organisms that are subject to similar evolutionary pressures due to their high relatedness and ecological similarities. However, the evolutionary dynamics of microorganisms allow for rapid change that may bring disparate lineages into conflict, especially if one lineage acquires a particular function of another via LGT. Thus, it becomes more straightforward to focus on ecological similarities, approximated by function defined at one or more levels of organization. How can we integrate functional similarities into a community analysis?

Functional overlap in spite of the apparent lack of an organismal core between samples of the same habitat has already provided convincing arguments in favor of a focus on ecological similarities. A recent example of this has been observed in the microbial communities associated with *Ulva australis*. Although only six OTUs were present in all sampled habitats (Burke *et al*., 2011b), and on average, 15% species similarity was seen between samples, and 70% functional similarity was observed across habitats. These functions spanned several categories such as motility, cell adhesion, biofilm formation, interaction with the host, and mechanisms of LGT (Burke *et al*., 2011a). The proteins involved in these functions in different samples were often phylogenetically distinct, suggesting functional convergence in disparate lineages. Such consistency of function has also been observed with regard to membrane proteins in the ocean. Patel *et al*. (2010) found correlations between transport proteins and inorganic chemical concentrations, but failed to find a corresponding link with species abundances. These functional profiles also correlated with environmental attributes including pollution, potentially allowing for these gene abundances to be utilized for predictions of such events. Barberán *et al*. (2012a) report that where 16S fails to differentiate marine microbial communities, genomic traits such as G+C content, genome size, and protein composition dramatically altered beta-diversity patterns and could better discriminate coastal from open-ocean samples and samples from the Atlantic, Pacific, and Indian oceans. Finally, the clinical significance of a shift from taxonomy-based to trait-based community ecology has already been demonstrated through the successful implementation of functional analyses and metagenomic linkage groups to discern microbiomes from type II diabetes patients and healthy individuals (Qin *et al*., 2012). The above examples all imply that within a given environment certain functional repertoires, defined either by collections of genes or by genomic properties, may be selected for and thus should be the focus of comparisons between habitats.

Although individual genes or *ab initio* generated combinations of genes may be predictive of phenotype or ecological role (e.g. MacDonald & Beiko, 2010), analyses that treat genes as uncorrelated entities will not always succeed in identifying important functional traits. For instance, Muegge *et al*. (2011) found that a diverse range of fecal microbial communities from different mammals clustered by diet type when 16S signatures were considered, but not when genes were summarized across all functional categories. Aggregation of genes into pathways and metabolic modules uses known associations between genes and allows for correction of incorrect predictions via gap filling and screening out of unlikely or redundant pathways (Ye & Doak, 2009; Abubucker *et al*., 2012). At the level of sequenced genomes, pathway- and module-based analyses have identified important functional correlations with periodontal disease (Kastenmüller *et al*., 2009). It is essential to choose the right trait definition for the question under scrutiny. Conserved traits are often assumed to track genome or organism evolution and thus may be expected to correlate with a wide range of genomic properties and functions (Langille *et al*., 2013). On the other hand, functional genes, pathways, or modules obtained from WGS confer information about a distinct set of traits that need not correlate with the phylogenetic relationships implied by 16S or other marker genes. To the extent that these different types of information can generate distinct and conflicting patterns, it may be worth combining them in an analysis.

How important are individual genes as mediators of community functions or interactions? Within a single cell, genes and gene products interact in a multitude of ways, for instance by direct chemical interaction, participation in the same biochemical pathway, transcriptional regulation, protein folding and refolding, and subcellular localization. These interactions place constraints on the evolutionary trajectory of genes: For example, the complexity hypothesis (Jain *et al*., 1999) predicts that genes whose products have many interactions are less likely to undergo LGT, suggesting lower LGT frequencies for ‘informational’ genes that tend to participate in large complexes such as the ribosome as compared with ‘operational’ genes with key metabolic and regulatory roles. This idea was made more explicit by Cohen *et al*. (2011) who showed that connectivity rather than function was the crucial determinant of gene transferability, which is consistent with the frequent transfer of aminoacyl-tRNA synthetases that are informational, but have few interaction partners in the cell (Woese *et al*., 2000; Andam & Gogarten, 2011).

In applying these insights from sequenced genomes to microbial communities, a central question is how these gene product interactions can mediate different types of interaction between community members. Gene loss and gene transfer according to the PGH and BQH along with the processes of duplication and substitution can lead to the formation of new community interactions; several such examples have been outlined above in the ecology of the dechlorinating communities, insect endosymbionts, biofilms, and SAR11. Cross-feeding is an obvious example of a microbial interaction, but some described or implied interactions are more complex and difficult to elucidate. For example, targeted studies of homologous genes from environmental samples have revealed remarkable and seemingly stable sequence diversity (Sabehi *et al*., 2003; Atamna-Ismaeel *et al*., 2008; Gabor *et al*., 2012), suggesting niche specialization (Bielawski *et al*., 2004) and the potential for rapid changes to nutrient sensitivity and host defense. Given the small amount of variation in these sequences and their presence in closely related strains that may possess identical 16S, the effects of these variations will depend on subtle differences in enzyme specificity or kinetics. Although transcription factors are unlikely to migrate between cells, there have been remarkable demonstrations of the ability of one taxon to induce significant changes in another, with dramatic ecological consequences. An example of this is seen in the lungs of cystic fibrosis patients that are subject to periodic exacerbations of the disease that lead to permanent declines in pulmonary function (Goss & Burns, 2007). With *Pseudomonas aeruginosa* as a primary pathogen of interest, researchers have identified a class of organisms including the *Streptococcus milleri* group, collectively termed ‘synergens’ that have neutral to positive impacts on hosts on their own, but increase mortality rates when combined with *P. aeruginosa* (Sibley & Surette, 2011). The specific interactions that induce the shift in pathogenic status remain to be elucidated, although transcriptional profiling under different association conditions will be highly informative (Duan *et al*., 2012).

## Genes as defining elements of networks and metacommunities

Microbial genomes typically contain many thousands of genes, many of which may mediate community interactions. A challenge in studying the ecological role of genes is the possibility that different genes may have opposing effects on organismal interactions. Furthermore, opposing selection processes at the gene vs. organism level would obscure the link between gene and community. A gene-centric view of communities will liberate microbial ecology from exclusively marker gene-driven approaches, but untangling the effects of different genes may require models that can accommodate distributional, phylogenetic, and selective information about those genes. Networks that incorporate these types of information would thus better reflect the dynamics of a microbial community, which may allow for variable taxonomic membership while retaining functional parameters. We turn our attention now to promising ecological frameworks based on gene exchange that may suit this purpose.

### Gene exchange communities

Up to now, we have considered microbial communities as defined by Konopka (2009) and others based on physical proximity of a set of organisms and the requirement that a set of organisms interact. However, LGT enables a different view of communities, where interactions between organisms are defined strictly on the basis of gene exchange (Jain *et al*., 2003; Skippington & Ragan, 2011). These gene exchange communities (GECs) are often represented by a graph or network structure with nodes signifying organisms or taxonomic groups and edges between nodes indicating evidence of LGT between a pair of groups. Additionally, edges can be *directed* if the identity of the donor and recipient can be reliably inferred and can also be *weighted* to reflect the extent of gene flow along a particular edge. DNA is sufficiently stable in the environment that GECs need not respect community or habitat boundaries and can span organisms that live in multiple habitats (Hooper *et al*., 2009; Holloway & Beiko, 2010; Smillie *et al*., 2011). Different types of DNA molecules including mobile phages and plasmids as well as chromosomes (Lima-Mendez *et al*., 2008; Halary *et al*., 2010) can also be considered separately in GECs. Such a *vehicle-centric* approach (Skippington & Ragan, 2011) can highlight the role of extrachromosomal elements in mediating LGT interactions between organisms.

### Metacommunities of genes

**Figure 5:**
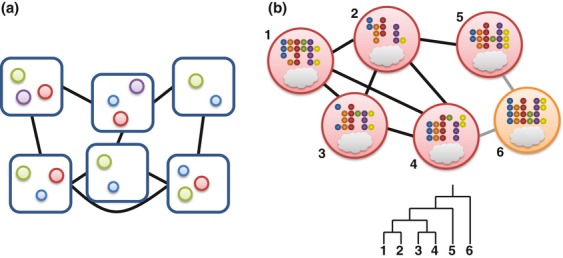
Metacommunity approaches in microbial community analysis. (a) Metacommunity of organisms, with locations as encompassing units, lines indicating migration pathways and different taxa indicated with color. (b) Metacommunity of genes, with organisms as units. Gray clouds represent the core genome, while colored circles indicate the presence or absence of different genes of different functional classes in the pan-genome. Lines indicate sharing of genes; gray lines connecting taxon 6 with other taxa represent reduced levels of LGT due to decreased efficiency of homologous recombination. The phylogenetic tree indicates the relationships between taxa based on a marker gene such as 16S.

Taking the idea of GECs one step further, the idea that genes mediate interactions suggests that analyses could consider communities of genes (once again in the ecological sense of ‘community’) in place of communities of organisms. An important recent development in microbial ecology is the application of metacommunity theory to microbial systems (Fig. 5a). A metacommunity was defined by Leibold *et al*. (2004) as ‘a set of local communities that are linked by dispersal of multiple potentially interacting species’. The definition does not require that species interact with one another and therefore encompasses all assemblages whether or not they satisfy our definition of communities. Modeling linkages allows the simultaneous consideration of dispersal, competition, and other processes and can be used to test hypotheses about the dynamics of assemblages and communities. In this framework, for instance, a person or an individual organ can be viewed as a ‘patch’ occupied by a microbial community, with assembly of that community mediated by the metacommunity in situations such as recovery from disturbance and invasive species (Costello *et al*., 2012). Declerck *et al*. (2013) applied metacommunity principles in the investigation of plankton community similarity, testing the effects of variable nutrient availability and dispersal rate in an outdoor mesocosm experiment. Varying nutrient availability did not affect the similarity between communities, although there was some evidence that nutrient addition did have a significant impact on community structure. However, even tiny amounts of dispersal between communities (corresponding to 0.009% of total volume) were sufficient to make these communities more similar to one another.

Such metacommunity analyses can yield significant insights into the dominant forces that influence microbial community structure. A promising extension of metacommunity theory is the explicit consideration of phylogenetic relatedness of the taxa in a study, which can highlight cases where the distribution of a taxon is restricted for historical reasons (Urban & Skelly, 2006; Cavender-Bares *et al*., 2009; Leibold *et al*., 2010). Malcom (2011) additionally considered the role of gene networks in shaping the phenotypes that allow organisms to compete in patches. Given the possibility of shifting the focus of a community investigation from species to genes (or functions, however defined), it may be worth investigating whether metacommunity models that were originally developed with taxonomic units in mind can be equally well applied to sets of genes (Fig. 5b). In this setting, one could view a microorganism (or a population of organisms) as a patch that is colonized by a set of genes, with an analogy between the historical biogeographic constraints of, for example, Leibold *et al*. (2010) and the phylogenetic histories (vertical or otherwise) of individual functional genes. The complete collection of genes in a population (roughly equivalent to the *pan-genome* of a species: Tettelin *et al*., 2005, 2008) could then be modeled as a metacommunity. ‘Selfish’ elements such as transposons and restriction/modification systems are good candidates for ecological analysis due to their high mobility. For example, Venner *et al*. (2009) reviewed ‘genome ecology’ approaches that treat transposable elements as interacting elements with host eukaryotic genomes as the niche, while Hooper *et al*. (2009) identified transposases, shared via LGT, which bridged multiple habitats. Restriction/modification systems are highly mobile, can impact on the fitness of their host in many ways, and interact with one another in ways that are often lethal to the cell (Kobayashi, 2001), and would likely map well into a metacommunity framework.

At the whole-genome level, an intriguing example of the application of community genetic ideas to microbial ecology is the exploration by Reno *et al*. (2009), who examined the biogeography of seven *Sulfolobus islandicus* genomes distributed across three locations in the United States and Russia. In this case, strong evidence of allopatric speciation was observed, with no gene flow between populations reflecting isolation of these thermoacidophilic organisms and little evidence of introgression from microorganisms outside of the Sulfolobales group. Here, dispersal limitation has essentially fractured the metacommunity of genes, such that genomes within each region still exhibit gene flow (equivalent to migration), but flow between regions is nonexistent. In contrast, the human microbiome is likely to show very different patterns, given the lack of barriers to dispersal and the demonstrated tendency of resident microorganisms to exchange genes (Salyers *et al*., 2004; Smillie *et al*., 2011; Meehan & Beiko, 2012). Here, we might expect multiple levels of gene flow (Skippington & Ragan, 2011), with exchange among closely related strains facilitated by homologous recombination and other processes. Moreover, ecology-driven LGT between more distant relatives could generate ‘higher-level units that resemble population-like assemblages’ (Andam & Gogarten, 2011). In both the *Sulfolobus* and human microbiome examples, a ‘metacommunity of genes’ framework is likely to yield insights into the ecological roles of genes, in tandem with the lineages that contain them.

## Defining and redefining the units of analysis

Having considered different ways of thinking about microbial communities in light of the evolutionary processes that shape the genomes of their constituents, we now consider the different definitions of ecological units that can be subjected to diversity analysis. Any ecological analysis of microorganisms will critically rest on the definition of the units or taxa to be counted, compared, and contrasted. Even before the advent of rapid DNA sequencing, a range of unit definitions emerged to balance taxonomy, phylogeny, and traits.

Taxonomic approaches have made use of OTUs, the application of which can be agnostic to the existence of taxonomic labels, thus allowing measurements of diversity and dissimilarity in the absence of a satisfactory taxonomic scheme. Because an OTU can be based upon any of a multitude of evolutionarily cohesive characters, including subregions of the 16S or other phylogenetic markers such as cpn60 (Case *et al*., 2007; Links *et al*., 2012), it allows for a range of markers to be utilized as the basis for diversity within a given sample or community (Hugenholtz *et al*., 1998; Schellenberg *et al*., 2011). A limitation of the OTU approach is that inferred groups for a fixed identity threshold will be different based on the choice of marker due to LGT or rate variation, potentially leading to different conclusions (Brousseau *et al*., 2001; Schellenberg *et al*., 2009). There is also sensitivity of the choice of method used to generate OTUs: Assignments can vary drastically depending on whether a 97% OTU is defined to require that a given sequence match all other sequences at this threshold or better (the ‘furthest neighbor’ approach) or whether it is sufficient that a given sequence matches any other sequence in the OTU (the ‘nearest neighbor’ approach). Phylogenetic approaches to diversity such as genome-based classification and inference among microorganisms (Klenk & Göker, 2010; Chan *et al*., 2012) address some of the limitations of OTU analysis (Lozupone *et al*., 2007). Still, they present a single picture of diversity that is dependent on a canonical hierarchical relationship.

A refinement of single marker diversity measures that still relies on phylogenetic cohesion is the concept of ecotypes (Cohan, 2002, 2006). This concept gives weight to common taxonomic properties between members of the same species, but differentiates based upon small changes in gene content or expression, which may allow for greater fitness within an ecological niche. Konstantinidis *et al*. (2006) defined ecotypes, based upon average nucleotide identity, to be members of a species that have accumulated a few small extra genetic elements or mutations for environmental adaptation, but otherwise preserve the overall genetic signature of the species. These approaches integrate the notion of OTU relatedness within a tight cluster, but allow for lineage segregation based upon small changes to the overall genetic background. Another departure from phylogenetically defined units is the concept of genovars, groups of strains that share distinct genetic content profiles (Porwollik *et al*., 2004) and form homologous recombination pools (Ahmed *et al*., 2012). These distinguishable groups that are below the level of species, but above the level of strain would likely form clusters within a taxonomy-free OTU study and would also group with other genovars in a species classification. Therefore, groupings such as genovars require a combination of functional and taxonomic diversity measures to understand their potential for pathogenicity or other functional features.

Pathogenicity and other phenotypic traits can also be shared collectively by members of a microbial assemblage or community and constitute a basis for classification. For example, pathogroups are polymicrobial biofilm communities that are integral to infections where the entire compilation of microorganisms contributes to a generalized pathogenic phenotype (Dowd *et al*., 2008). Such polymicrobial biofilms demonstrate diverse community properties, which allow for invasion of host tissue and subsequent cell adherence in tandem with passive antibiotic resistance and metabolic handovers (Wolcott *et al*., 2013). These biofilms are composed of highly integrated yet diverse parts with active mechanisms of recruitment to ensure such variety is achieved. Coordinated yet diverse communities have also been observed in nonpathogenic settings such as dental plaques (McBride & Van der Hoeven, 1981; Kuramitsu *et al*., 2007). These biofilms are clear examples of where the diversity of the community matters and interplay between such diverse members, be they of differing species, strains, or levels in between, form the basis of a community and directed associated interactions with the habitat. Studies based upon single marker genes in an organism-by-organism setting will not elucidate such patterns, because a coupling of functional trait-based approaches and taxonomic contexts is required to observe fine-grained diversity and related community functionality. This coupling can inform experimental procedures for studying such communities and their interaction networks.

Another approach that can link taxonomically disparate organisms is the use of phylogenetic networks (Hilario & Gogarten, 1993; Beiko *et al*., 2005; Kunin *et al*., 2005; Beiko, 2011; Dagan, 2011; Parks & Beiko, 2012), rather than phylogenetic trees, as the basis for phylogenetic beta-diversity. Such networks can represent the uncertainty in a phylogenetic tree (e.g. Parks & Beiko, 2012) or show conflicting similarity relationships as derived from a sampling of many genes rather than a single phylogenetic marker. The latter type of network could modify diversity values by downweighting the ecological differences among organisms that participate in the same gene exchange community, for instance by tracing the shortest path between a pair of taxa instead of the canonical relationship derived from a marker gene.

## Testing the community hypothesis

Because our working definition presents microbial communities as a hypothesis rather than a mere set of observations, experimental and computational approaches need to be designed with communities in mind. In Box 1, we outline protocols that assess the growth response of microorganisms, target the metabolites they produce, and enable a genomic view of organismal interactions. Given the central importance of sequence data in microbiomics, the remainder of this section is focused on emerging methods that can target the question of microbial communities.

### Computational approaches for marker gene and metagenomic data

**Figure 6:**
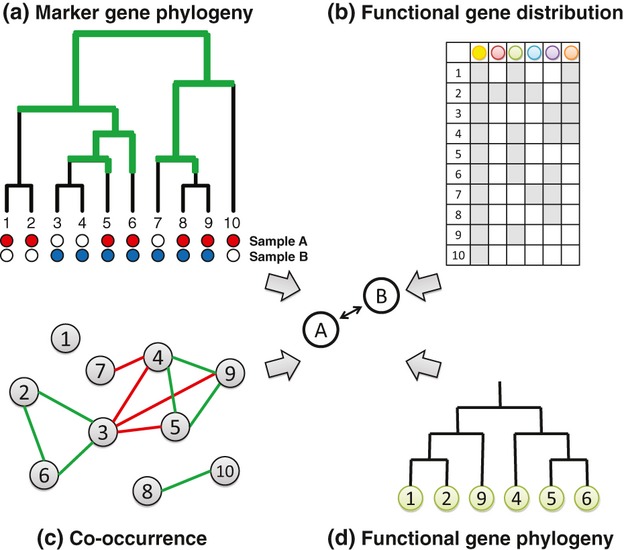
Computing diversity (expressed here as the dissimilarity between samples A and B) with multiple types of data. (a) A phylogeny of marker genes, which serves as the basis for most studies of microbial beta-diversity. (b) The distribution (i.e. phylogenetic profile) of different classes of genes can highlight associations that do not necessarily coincide with the phylogeny in (a), suggesting evolutionary and possibly functional connections between more distant taxa. (c) Co-occurrence networks display positively and negatively correlated sets of taxa, highlighting possible species sorting effects and functionally equivalent or similar taxa. Such taxa could contribute relatively little to overall beta-diversity. (d) Like the distributions in (b), phylogenies of nonmarker genes can recapitulate the dispersal of genes across a set of taxa in a nonvertical manner and identify taxa that are more functionally similar than their marker gene phylogeny would suggest.

Bioinformatics, central to the analysis of microbial communities, will benefit from the development of new descriptive standards such as MIMARKS to describe marker genes (Yilmaz *et al*., 2011) and the emergence of reference databases and formats that aim to adhere to these standards (Gilbert *et al*., 2010b; Ivanova *et al*., 2010; McDonald *et al*., 2012). An important first question is how the current taxonomic and phylogenetic strategies for inferring community structure can be augmented with additional information about function and distribution (Fig. 6). Martiny *et al*. (2009) demonstrated the value of using different OTU thresholds to discover different environmental correlates in samples of *P. marinus*. In using supervised learning approaches to classify microbiome samples based on OTU abundance, Knights *et al*. (2011) found similar performance across a wide range of OTU thresholds and suggested that ‘hybrid models using several levels of phylogenetic binning will outperform those constrained to any one bin size, and this is certainly an area that requires further research’. Large reference databases such as the Earth Microbiome Project associate marker gene distributions with a wide range of habitat and temporal information; these resources will provide a rich reference set against which new data sets can be compared.

While phylogenetic proximity is the most commonly used surrogate for ecological similarity, as evidenced by the proliferation of studies using OTU-based and phylogenetic beta-diversity measures to infer community similarity, these new sources of information suggest new approaches based on habitat similarity and/or co-occurrence. Recent work suggests that molecular functions are in many cases predictable from marker gene information, if phylogenetically close reference genome sequences are available (Langille *et al*., 2013; Martiny *et al*., 2013). Although the genome evolution processes described above can lead to different degrees of predictability for different types of molecular function and for different lineages, these observations suggest that OTU analyses can be enriched with functional information to produce better definitions of ecological units and predictions of ecological roles (Fuhrman, 2012). Given the value and predictive power of phylogenetic marker genes, especially at the genus and species level, we anticipate that the *implicit* functional information conveyed by marker genes, along with *explicit* information from functional genes (e.g. functional gene networks: Bittner *et al*., 2010) and habitat distribution (e.g. co-occurrence networks: Chaffron *et al*., 2010; Barberán *et al*., 2012b; Faust *et al*., 2012), will prove a powerful combination in the ecological analysis of microorganisms (Fig. 6). For example, Layeghifard *et al*. (2012) made the connection between phylogenetic networks and species dispersal, using the network approach of Boc *et al*. (2010) for inferring networks from genetic data, to reconstruct migration networks from geographic and biodiversity information.

**Box 1. Experimental approaches to investigating assemblages and communities**

Although sequencing of environmental DNA currently dominates the study of microbial assemblages and communities, a variety of allied technologies are required to determine what is transcribed (metatranscriptomics: Poretsky *et al*., 2005; Leininger *et al*., 2006; Moran *et al*., 2013), the proteins present in a sample (metaproteomics: Rodríguez-Valera, 2004; Wilmes & Bond, 2004; Ram *et al*., 2005), and what metabolites are produced (metabolomics: Weckwerth, 2003). Metaproteomics (Wilmes & Bond, 2004) can reveal new functional genes and metabolic pathways in a sample. Ram *et al*. (2005) used ‘shotgun’ mass spectrometry approaches to identify correlations between organismal abundance and level of protein expression and highlight the apparent importance of hypothetical proteins as well as proteins involved in refolding (e.g. chaperones) and oxidative stress. Metaproteomics has also been used to identify strain-level variation in *Candidatus* ‘Accumulibacter phosphatis’ protein expression in enhanced biological phosphorus removal (EBPR) communities (Wilmes *et al*., 2008a) and directly link these proteins to EBPR metabolic processes (Wilmes *et al*., 2008b). These approaches have been combined to further characterize microbial communities (e.g. Gilbert *et al*., 2010a; Teeling *et al*., 2012; Yu & Zhang, 2012). While none of these approaches can necessarily identify the precise nature of an interaction, they can be used to gauge the impact of shifts in environment or assemblage on the function of an organism.

Single-cell isolation and sorting techniques can be used to subdivide communities and facilitate genomics, proteomics, and transcriptomics of a select group of cells to gain insight about community ecology (Müller *et al*., 2012). For example, Jehmlich *et al*. (2010) used cell-sorting techniques to separate *E. coli* K-12 from *Pseudomonas putida* KT2440 in a mixed culture of 5 × 10^6^ cells and applied proteomics to identify proteins that were expressed in each subpopulation. The sequenced genomes of five cells of the *Verrucomicrobia* obtained through single-cell isolation from bacterioplankton communities revealed that these organisms are capable of hydrolysis of a wide variety of polysaccharides, which is important in bacterioplankton communities (Martinez-Garcia *et al*., 2012).

Potential interactions in a set of microorganisms can be assessed by measuring the impact they have on microbial growth rates in culture. Experimental systems such as microcosms, chemostats, and mixed cultures have produced a large body of knowledge about the evolution, ecology, and physiology of organisms and communities in a wide variety of natural and artificial habitats. Trzesicka-Mlynarz & Ward (1995) discovered the importance of mixed cultures in degradation of polycyclic aromatic hydrocarbons: Mixed cultures of *P. putida*, an unknown flavobacterium, and *P. aeruginosa* degraded a wider range of polycyclic aromatic hydrocarbons, relative to pure cultures of each bacterium. Kuenen (1983) examined the role of competition between specialists and a generalist in a mixed culture: Specialists *Thiobacillus neopolitanus* and *Spirillum* G7 with generalist *Thiobacillus* A2 were placed in various growth media; *Thiobacillus* A2 outcompeted the other strains on mixed media, while the specialists were more successful on specialized media. Sher *et al*. (2011) discovered that co-cultures including one of two closely related strains of the photoautotroph *P. marinus* and one of 344 strains of various heterotrophic bacteria enhanced growth curves in a manner dependent on the relatedness of the *P. marinus* strains. As with transcriptomic and other types of data described above, it may not be possible to deduce the exact nature of an interaction from co-culture experiments alone.

Historical limitations to characterization of community metabolism are being remedied with new laboratory techniques. Available techniques include stable isotope probing (SIP) for culture-independent tracking of molecules through microbial communities and their members (Kreuzer-Martin, 2007) and imaging mass spectrometry (Watrous *et al*., 2011) for collecting direct evidence of chemical interaction between community members. DNA stable isotope probing (DNA-SIP) combines stable isotope tagging to molecules to allow the identification and function of organisms that metabolize the tagged molecules (Chen & Murrell, 2010). Schloss & Handelsman (2003) suggested the use of DNA-SIP to subdivide microbial communities for metagenomic sequencing, while a proof of concept was used to isolate large DNA fragments from uncultured soil bacteria (Dumont *et al*., 2006). Other work used DNA-SIP and metagenomics to dramatically increase the chance of finding specific functional genes from metagenomes (Knietsch *et al*., 2003; Sul *et al*., 2009).

Fine-scale understanding about the interaction between any two organisms can also be obtained through insertional mutagenesis and depletion (iMAD), which combines bacterial mutagenesis and RNA interference. Using iMAD, the dynamics of interaction between *Legionella pneumophila* and its host was resolved, revealing the network of proteins that are required for intracellular growth of *L. pneumophila* (O’Connor *et al*., 2012). Desorption electrospray ionization (DESI) has verified already known metabolic interactions between competing *Bacillus subtilis* and *Streptomyces coelicolor* (Watrous *et al*., 2010), and nano-DESI has been used to examine the molecular networks of living colonies, including the possible identities of unknown metabolites through time (Watrous *et al*., 2012). Nano-DESI could be potentially useful in reconstructing the metabolism of a community of organisms (i.e. a multispecies metabolic network), establishing alternative organismal physiologies, and when combined with sequencing, help in the verification of gene function.

**Figure 7:**
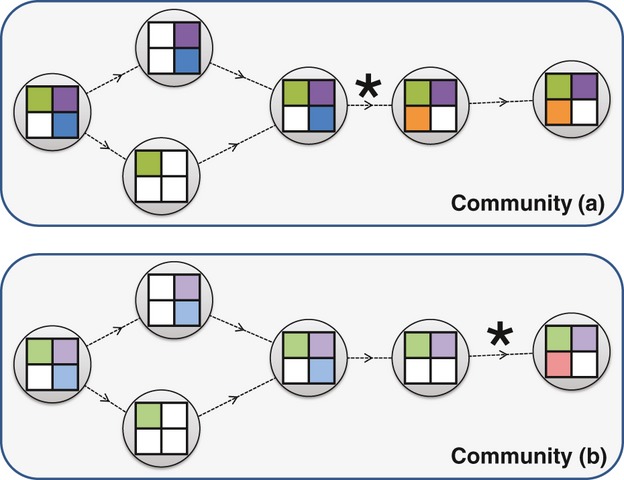
Identifying handoff points in metagenome samples. Steps in a directed, branching metabolic pathway are shown, with colored squares indicating the presence of a given reaction in different members of a microbial community. Some organisms such as the blue individuals in both communities encode only the first few steps of the pathway and do not require the products of later steps. However, handoff points (indicated with ‘*’) are steps where an organism depends on other members of the community for synthesis of a particular metabolite. The handoff point locations differ for the orange taxon in (a) and the pink taxon in (b), possibly due to different combinations of LGT and gene loss in the impacted organisms.

Another promising approach is to construct more explicit models of microbial and community function to address the partitioning of functions across taxa in a sample. Our understanding of metagenomic samples is constrained by the large number of hypothetical proteins for which reliable functions are not available (Galperin & Koonin, 2004; Ellrott *et al*., 2010) and the high degree of misannotation of some functional families of proteins (Schnoes *et al*., 2009; Radivojac *et al*., 2013). However, educated guesses about protein function can be made based on cues such as phylogeny, genetic linkage, subcellular localization, and metabolic pathway cohesion (Yu *et al*., 2010; Engelhardt *et al*., 2011; Yelton *et al*., 2011), and these predictions can be improved through computational means (Chen *et al*., 2013) and tested experimentally (Mirete *et al*., 2007; Yamada *et al*., 2012). Predicted genes in metagenomes can be subjected to both functional and taxonomic assignment, to divide the functional profile of the microbiome by organism or lineage. Although taxonomic assignment is imperfect (McHardy & Rigoutsos, 2007; MacDonald *et al*., 2012) especially when reference taxa are lacking or multiple strains are present, this information can nonetheless be used to determine which organisms are providing which crucial functions in a community (Hug *et al*., 2012). Ideally, such analyses can reveal metabolic pathway discontinuities or ‘handoff points’ that correspond to syntrophic or other types of association (Fig. 7).

Systems biology approaches have been developed to model the flux of metabolites within and between community members. Existing models can struggle with the number of steps and the complexity of interactions involved, but simplified approaches that focus on particular functions of interest have yielded testable predictions (Stolyar *et al*., 2007; Salimi *et al*., 2010; Zhuang *et al*., 2012; Zomorrodi & Maranas, 2012). Although interactions predicted from metabolic networks and models still need to be tested through experimentation, they will quickly be able to highlight potential positive and negative interactions between microorganisms based on metagenomic data. Enumerating many different types of communities in this fashion will reveal which types of dependencies (for instance, cofactor synthesis, carbohydrate degradation, and dependence on others for oxygen scavenging) have emerged most often.

### An evolutionary context for microbial communities

The evolutionary trajectory of genomes can inform us about processes of community formation and specialization of microorganisms. The interactions between organisms in a putative community can be probed using the methods described above, but comparisons against completely sequenced reference genomes highlight differences that may reflect recent community evolution, including evidence of ‘public goods’ sharing or gene losses according to the BQH. The application of metacommunity theory to genes in a set of metagenomes in concert with closely related sequenced genomes would produce novel ecological views of the metagenome in relation to habitat and the partitioning of genes among organisms. This approach extends ideas already developed in the examination of shared gene pools in environmental organisms such as *S. islandicus* (Reno *et al*., 2009) and lineages such as *Listeria* that include both pathogenic and environmental isolates (Dunn *et al*., 2009). Johnson & Winquist (2011) proposed that changes in hygiene have impacted on the biogeography of gut bacteria, effectively altering the metacommunity structure by increasing the fragmentation of communities. Such shifts would undoubtedly impact LGT regimes as well.

Evolutionary pressures manifested at the gene level will also shed light on the role of different genes in a community. In *M. leprae*, the ratio of nonsynonymous to synonymous mutations (dN/dS) can be used to identify genes under reduced selection pressure, indicating possible losses of function that will ultimately lead to deletion from the genome. Calculated dN/dS ratios significantly > 1.0 constitute evidence for positive selection and may indicate rapid adaptation to a new or changing habitat (Ohta, 1992). Increased rates of nonsynonymous substitution have also been observed in reduced endosymbionts (Kuo *et al*., 2009) and have been interpreted to reflect reduced effective population sizes in pathogens (Warnecke & Rocha, 2011). In the human microbiome, Schloissnig *et al*. (2013) found no genes with strong evidence of positive selection, but identified antimicrobial genes and bile salt hydrolase as the best candidates for undergoing localized and rapid nonsynonymous change. Conversely, an examination of iron uptake genes from the Global Ocean Sampling database identified a number of iron uptake and metabolism genes that showed evidence of positive selection, suggesting adaptations to nutrient limitation that echo the quantitative study of Patel *et al*. (2010). Complementing the study of substitution rates is the search for evidence of genetic recombination within genes. Recombination and LGT have been shown to replace parts of genes as well as entire genes (Schouls *et al*., 2003; Chan *et al*., 2009, 2011), and recombination within some types of genes such as those encoding surface proteins could be interpreted as a modifying force akin to positive selection (Hollingshead *et al*., 2000; Baldo *et al*., 2010).

## Conclusion

The preeminent questions in microbial ecology today can be traced back to the original diversity surveys of van Leeuwenhoek and ecological studies of succession in the late 17th century. The intervening 300 years have provided evidence that microorganisms are alive and abundant in every setting on Earth, can cause disease, possess genetic material, are central to nutrient cycling, and can evolve quickly to adapt to new challenges and opportunities. With every significant new technique developed to study microorganisms, the predominant thinking of earlier periods has been overturned, reshaping the debate about their fundamental nature and the roles they play (Sapp, 2005). Affordable and fast DNA sequencing drives the latest revolution, and the rate at which genomic (including metagenomic) data are accumulating easily outstrips our capacity to thoroughly analyze and reason about microbial communities. Even where informed analysis is possible, important conclusions can be entirely dependent on the statistical and computational tools that are applied and the choice of approach used to categorize diversity and function. A striking example of this dependency is the claim that sets of microorganisms in the human gut can be classified into ‘enterotypes’ that differ qualitatively in their composition and function. Arumugam *et al*. (2011) claimed the existence of enterotypes based on the observation that the taxonomic profile associated with any gut sample could be assigned to one of three enterotypes. However, other evidence suggests that enterotypes are a product of the analytical methods used to analyze the data (MacDonald *et al*., 2012; Yatsunenko *et al*., 2012; Koren *et al*., 2013) and that diversity is best represented as a gradient rather than a finite and small number of discrete states (Jeffery *et al*., 2012; Yatsunenko *et al*., 2012). Although more data (larger samples, more individuals, time series) are essential to resolving this question, microbial community theory will have a central role to play as well, because the degree of interactions will influence the tendency of communities to behave like discrete entities or gradients.

Even microbial communities with low apparent diversity present a multitude of challenges. The extreme acid mine drainage biofilm environment has been probed extensively using sequence-based and complementary approaches (Tyson *et al*., 2004; Belnap *et al*., 2010; Jiao *et al*., 2011). While the community is dominated by a narrow range of lineages including *Leptospirillum* and *Ferroplasma* (Tyson *et al*., 2004), population-level analysis has revealed extensive sequence diversity and evidence of LGT among closely related strains (Allen *et al*., 2007; Eppley *et al*., 2007), rare lineages of tiny Archaea (Baker *et al*., 2006), multiple lineages of acidophilic eukaryotes, some of which act as hosts to bacterial endosymbionts (Baker *et al*., 2003, 2009), and complex bacteriophage interactions with rapid turnover of CRISPR sequences (Andersson & Banfield, 2008). Modeling the evolutionary and ecological interactions within even this ‘simple’ community is a daunting task; a challenge that is only amplified in less extreme environments that have more niches, higher richness and diversity, and greater disturbances including increased competition through dispersal. Clearly, lineage-based analyses alone are insufficient to the task of modeling communities.

A question that is highly pertinent to microbial ecology was posed by Webb *et al*. (2010): ‘Can ecological performance generally be predicted by a single or just a few traits or are many traits required?’ Currently, our knowledge of many microbiomes is based on a single trait, the 16S, which is present in all prokaryotes, but conveys no direct evidence of ecological differences. Using genomes and metagenomes shifts the balance from too few to too many traits, and analyzing all genes indiscriminately will lose important functional differences in an ocean of largely uninformative functional information. For this reason, we see a great deal of promise in approaches that fuse phylogenetic information (which can serve as a proxy for many traits that are conserved at low-to-medium taxonomic ranks) with specific functions of interest and information about habitat distribution (with species sorting as another imperfect proxy for function). In concert with these approaches to diversity, we believe that treating genes as ecological agents will yield vital new insights into the question of whether shifts in taxonomic composition necessarily imply shifts in community function. If different organisms in different communities are fulfilling the same roles, it is more likely that the communities differ in their taxonomic composition due to stochastic processes such as founder effects or density-dependent effects such as phage predation. Conversely, functional differences – even in only a few critical pathways – could reflect subtle, but important habitat differences and dramatically altered ecosystem services. This focus on functional attributes, and the corresponding view of genes as agents that confer selective advantages, echoes the ‘selfish gene’ hypothesis of Dawkins (1976), but allows genes even more freedom to follow distinct evolutionary trajectories thanks to LGT. However, identifying crucial functional differences is a daunting challenge given the confounding effects of hypothetical proteins and incorrect functional annotations. Identified pathways may also be irrelevant if an organism either is incapable of expressing that pathway or is merely a ‘tourist’ that is isolated from interactions with the host or other observed microorganisms. Replicated experiments and the use of complementary approaches such as transcriptomics will provide partial solutions to these problems (Knight *et al*., 2012).

Microbiomics is already being applied in a wide range of settings. The preeminent example in human health is the use of fecal transplants containing a defined mixture of nonpathogenic organisms to cure *Clostridium difficile* infection (Lawley *et al*., 2012; Petrof *et al*., 2013). Community construction, enrichment, and amendment are being used to optimize many microbial bioremediation processes (Duhamel *et al*., 2004; Brune & Bayer, 2012; Mikesková *et al*., 2012; Patel *et al*., 2012), while environmental monitoring has revealed startling shifts in the Arctic Ocean microbiota, a potential harbinger of further shifts as the state of the region changes (Comeau *et al*., 2011). A deeper understanding of the relationships and dependencies between microorganisms in a community will recast central questions in microbiology from ‘Who is there?’ and ‘What are they doing?’ to ‘How will they respond?’ New techniques for community analysis and modeling will influence the design of experiments and suggest interventions to produce desirable changes in microbial community function. With this will come the realization of the full potential of microbiomics.
